# Outline, Divergence Times, and Phylogenetic Analyses of Trechisporales (Agaricomycetes, Basidiomycota)

**DOI:** 10.3389/fmicb.2022.818358

**Published:** 2022-04-25

**Authors:** Zhan-Bo Liu, Ying-Da Wu, Heng Zhao, Ya-Ping Lian, Ya-Rong Wang, Chao-Ge Wang, Wei-Lin Mao, Yuan Yuan

**Affiliations:** ^1^School of Ecology and Nature Conservation, Beijing Forestry University, Beijing, China; ^2^Key Laboratory of Forest and Grassland Fire Risk Prevention, Ministry of Emergency Management, China Fire and Rescue Institute, Beijing, China

**Keywords:** Hydnodontaceae, phylogenetic analysis, *Trechispora*, taxonomy, wood-rotting fungi

## Abstract

Phylogenetic analyses inferred from the nuc rDNA ITS1-5.8S-ITS2 (ITS) data set and the combined 2-locus data set [5.8S + nuc 28S rDNA (nLSU)] of taxa of Trechisporales around the world show that *Sistotremastrum* family forms a monophyletic lineage within Trechisporales. Bayesian evolutionary and divergence time analyses on two data sets of 5.8S and nLSU sequences indicate an ancient divergence of *Sistotremastrum* family from Hydnodontaceae during the Triassic period (224.25 Mya). *Sistotremastrum* family is characterized by resupinate and thin basidiomata, smooth, verruculose, or odontoid-semiporoid hymenophore, a monomitic hyphal structure, and generative hyphae bearing clamp connections, the presence of cystidia and hyphidia in some species, thin-walled, smooth, inamyloid, and acyanophilous basidiospores. In addition, four new species, namely, *Trechispora dentata*, *Trechispora dimitiella*, *Trechispora fragilis*, and *Trechispora laevispora*, are described and illustrated. In addition, three new combinations, namely, *Brevicellicium daweishanense*, *Brevicellicium xanthum*, and *Sertulicium limonadense*, are also proposed.

## Introduction

Trechisporales K.H. Larss. was established by Hibbett et al. (2007). Most species in this order are corticioid fungi with smooth, grandinioid, odontioid, or hydnoid hymenophores, and others are polypores. All species have a monomitic or dimitic hyphal system with generative hyphae bearing clamp connections, and many species have rhizomorphs (mycelial cords) (Larsson, 2007).

At present, there is only an acknowledged and a named family belonging to Trechisporales, i.e., Hydnodontaceae Jülich. Hydnodontaceae contains 11 genera now, namely, *Brevicellicium* K.H. Larss. and Hjortstam, *Dextrinocystis* Gilb. and M. Blackw., *Fibrodontia* Parmasto, *Pteridomyces* Jülich, *Luellia* K.H. Larss. and Hjortstam, *Porpomyces* Jülich, *Scytinopogon* Singer, *Subulicystidium* Parmasto, *Suillosporium* Pouzar, *Trechispora* P. Karst., and *Tubulicium* Oberw (Larsson, 2007; Spirin et al., 2021).

*Trechispora* is the genus type of Trechisporales and Hydnodontaceae. It is the largest genus in this order, with more than 50 accepted species (Meiras-Ottoni et al., 2021; Zhao and Zhao, 2021). Identification keys for *Trechispora* species recorded in China and Brazil have been provided by some fungal taxonomists (Chikowski et al., 2020; Meiras-Ottoni et al., 2021; Zong et al., 2021). *Trechispora* was typified with *Trechispora onusta* P. Karst. [= *Trechispora hymenocystis* (Berk. and Broome) K.H. Larss.] (Karsten, 1890). It is characterized by the resupinate basidiomata (a few species have stipitate, flabellate, and effused–reflexed basidiomata) with smooth grandinioid, odontioid, hydnoid, or poroid hymenophores, a monomitic or dimitic hyphal structure with clamped generative hyphae and smooth to verrucose or aculeate basidiospores (Larsson, 1992; Larsson et al., 2004). Most species in *Trechispora* are soil-dwelling (Larsson et al., 2004). One remarkable character is the presence of ampullate septa on the subicular and especially on some hyphae of the mycelial cords. Above all, ampullate septa are only known from *Scytinopogon*, *Trechispora*, and *Porpomyces mucidus* (Pers.) Jülich within Trechisporales (Furtado et al., 2021; Meiras-Ottoni et al., 2021).

Larsson (2007) used the term “*Sistotremastrum* family” for the first time to accommodate *Sistotremastrum suecicum* Litsch. ex J. Erikss. and *Sistotremastrum niveocremeum* [= *Sertulicium niveocremeum* (Höhn. and Litsch.) Spirin and K.H. Larss.]. Since then, “*Sistotremastrum* family” has been adopted by some taxonomists (Telleria et al., 2013; Liu et al., 2019). In this work, the phylogeny of Trechisporales is carried out based on combined 5.8S + nLSU sequences. In addition, Bayesian evolutionary and divergence time analyses are also carried out to indicate the divergence time of Trechisporales, Hydnodontaceae, and *Sistotremastrum* family. We outline the *Sistotremastrum* family and discuss the difference between Hydnodontaceae and *Sistotremastrum* family.

During investigations on the diversity of wood-rotting fungi, seven resupinate specimens were collected from China and Malaysia. Their morphology corresponds to the concept of *Trechispora*. To confirm their affinity, phylogenetic analyses based on the ITS sequences are carried out. Both morphological characteristics and molecular evidence demonstrate that these seven resupinate specimens represent the four new species of Trechispora.

In addition, we downloaded the type sequences of *Trechispora daweishanensis* C.L. Zhao, *Trechispora xantha* C.L. Zhao, and *Sistotremastrum limonadense* G. Gruhn and P. Alvarado from GenBank. We also studied the type specimens of *T. daweishanensis* and *T. xantha*. In conclusion, *T. daweishanensis* and *T. xantha* were transferred to *Brevicellicium*, while *S. limonadense* was transferred to *Sertulicium.*

## Materials and Methods

### Morphological Studies

Macro-morphological descriptions are based on field notes and dry herbarium specimens. Microscopic structures are photographed using a Nikon Digital Sight DS-L3 (Japan) or Leica ICC50 HD (Japan) camera. Microscopic measurements are made from slide preparations of dry tissues stained with 1% Phloxine B (C_20_H_4_Br_4_Cl_2_K_2_O_5_) (Fan et al., 2021). We also use other reagents, such as Cotton Blue and Melzer’s reagent following Dai’s (2010) study. Spore measurements include both with ornamentation and without ornamentation. The following abbreviations are used: KOH = 5% potassium hydroxide; CB = Cotton Blue; CB(+) = weakly cyanophilous; CB− = acyanophilous in Cotton Blue; IKI = Melzer’s reagent; IKI− = neither amyloid nor dextrinoid in Melzer’s reagent; *L* = mean spore length (arithmetic average of all spores including ornamentation); *W* = mean spore width (arithmetic average of all spores including ornamentation); *Q* = a variation in the *L*/*W* ratios between the specimens studied; *L*′ = mean spore length (arithmetic average of all spores excluding ornamentation); *W*′ = mean spore width (arithmetic average of all spores excluding ornamentation); *Q*′ = a variation in the *L*′/*W*′ ratios between the specimens studied; *n* (*a*/*b*) = the number of spores (*a*) measured from a given number of specimens (*b*). When presenting spore size variation, 5% of measurements are excluded from each end of the range and these values are given in parentheses. Special color terms follow Petersen (1996). Herbarium abbreviations follow Thiers (2018). The studied specimens are deposited at the herbarium of the Institute of Microbiology, Beijing Forestry University (BJFC), and the herbarium of Southwest Forestry University (SWFC).

### DNA Extraction, Polymerase Chain Reaction Amplification, and Sequencing

Total genomic DNA from the dried specimens is extracted by a CTAB rapid plant genome extraction kit (Aidlab Biotechnologies Company Limited, Beijing, China) according to the manufacturer’s instructions with some modifications (Liu and Yuan, 2020; Du et al., 2021). The ITS regions are amplified with the primers ITS4 and ITS5 (White et al., 1990). The nLSU regions are amplified with the primers LR0R and LR7 (Vilgalys and Hester, 1990).

The polymerase chain reaction (PCR) procedure for ITS is as follows: initial denaturation at 95°C for 3 min, followed by 35 cycles at 94°C for 40 s, 58°C for 45 s, and 72°C for 1 min, and a final extension of 72°C for 10 min. The PCR procedure for nLSU was as follows: initial denaturation at 94°C for 1 min, followed by 35 cycles at 94°C for 30 s, 48°C for 1 min, and 72°C for 1.5 min, and a final extension of 72°C for 10 min (Zhao et al., 2015; Liu and Dai, 2021). The PCR products are purified and sequenced in the Beijing Genomics Institute, China, with the same primers used in the PCR reactions.

### Phylogenetic Analyses

Two combined matrices, an ITS1-5.8S-ITS2 (ITS) data set and a two-gene data set (5.8S + nLSU), are used for phylogenetic analyses. Phylogenetic analyses are performed with maximum likelihood (ML), maximum parsimony (MP), and Bayesian inference (BI) methods in the ITS data set. Phylogenetic analyses are performed with ML and BI methods in the combined two-gene data set (5.8S + nLSU). Species and strain sequences are adopted partly from 28S- and ITS-based tree topologies established by Meiras-Ottoni et al. (2021) and Spirin et al. (2021). New sequences generated in this study, along with reference sequences retrieved from GenBank (Table 1), are aligned by MAFFT 7 (Katoh et al., 2019^1^) using the “G-INS-i” strategy and manually adjusted in BioEdit (Hall, 1999). Unreliably aligned sections are removed before analyses and attempts are made to manually inspect and improve alignment. The data matrix is edited in Mesquite v3.70 software (Maddison and Maddison, 2021). The sequence alignment is deposited at TreeBase (submission ID 29141 and 29142). Sequences of *Auricularia* sp., *Exidia recisa* (Ditmar) Fr., and *Exidiopsis calcea* (Pers.) K. Wells are included in phylogenetic analyses. They belong to another order, Auriculariales Bromhead. The order is close to Trechisporales (Sulistyo et al., 2021). We add these three sequences in the combined two-gene data set (5.8S + nLSU) to demonstrate that Trechisporales forms a strongly supported sister clade to Auriculariales. Sequences of *Hyphodontia floccosa* (Bourdot and Galzin) J. Erikss. and *Hyphodontia subalutacea* (P. Karst.) J. Erikss. in Hymenochaetales Oberw. obtained from GenBank are used as outgroups to root trees in the 5.8S + nLSU analysis. Two sequences of *Brevicellicium atlanticum* Melo, Tellería, M. Dueñas and M.P. Martín obtained from GenBank are used as outgroups to root trees in the ITS analysis.

**TABLE 1:** Information of taxa used in phylogenetic analyses.

Species	Collector ID (herbarium ID)	GenBank accession no.	
		ITS	nLSU
*Auricularia* sp.	PBM 2295	DQ200918	AY634277
*Brevicellicium atlanticum*	LISU 178566 (holotype)	NR_119820	HE963774
*Brevicellicium atlanticum*	LISU 178590	HE963775	HE963776
***Brevicellicium daweishanense***	**CLZhao 18255 (SWFC)**	**MW302338**	**MW293867**
***Brevicellicium daweishanense***	**CLZhao 17860 (SWFC, holotype)**	**MW302337**	**MW293866**
*Brevicellicium exile*	MA-Fungi 26554 (holotype)	HE963777	HE963778
*Brevicellicium olivascens*	KHL 8571 (GB)	HE963792	HE963793
*Brevicellicium olivascens*	MA-Fungi 23496	HE963787	HE963788
***Brevicellicium xanthum***	**CLZhao 17781 (SWFC)**	**MW302340**	**MW293869**
***Brevicellicium xanthum***	**CLZhao 2632 (SWFC, holotype)**	**MW302339**	**MW293868**
*Dextrinocystis calamicola*	He 5700 (BJFC)	MK204534	MK204547
*Dextrinocystis calamicola*	He 5693 (BJFC)	MK204533	MK204546
*Exidia recisa*	EL 15-98 (GB)	AF347112	AF347112
*Exidiopsis calcea*	MW 331	AF291280	AF291326
*Fibrodontia alba*	TNM F24944 (holotype)	KC928274	KC928275
*Fibrodontia gossypina*	AFTOL-ID 599	DQ249274	AY646100
*Hyphodontia floccosa*	Berglund 150-02 (GB)	DQ873618	DQ873617
*Hyphodontia subalutacea*	GEL2196 (KAS)	DQ340341	DQ340362
*Porpomyces mucidus*	Dai 12692 (BJFC)	KT157833	KT157838
*Porpomyces submucidus*	Cui 5183 (BJFC)	KU509521	KT152145
*Pteridomyces galzinii*	GB0150230	LR694188	LR694210
*Pteridomyces galzinii*	Bernicchia 8122 (GB)	MN937559	MN937559
*Scytinopogon angulisporus*	TFB13611	–	JQ684661
*Scytinopogon chartaceum*	FLOR56185	MK458775	–
*Scytinopogon pallescens*	He 5192 (BJFC)	–	MK204553
*Sertulicium chilense*	MA-Fungi 86368 (holotype)	HG315521	–
*Sertulicium granuliferum*	He 3338	MK204552	MK204540
*Sertulicium jacksonii*	Spirin 10425 (H)	MN987943	MN987943
*Sertulicium lateclavigerum*	LY 13467	MG913225	–
***Sertulicium limonadense***	**LIP 0001683 (holotype)**	**MT180981**	**MT180978**
***Sertulicium limonadense***	**He 6276 (BJFC)**	**OK298489** *	**OK298947** *
*Sertulicium niveocremeum*	KHL13727 (GB)	MN937563	MN937563
*Sertulicium vernale*	Soderholm 3886 (H, holotype)	MT002311	MT664174
*Sistotremastrum aculeatum*	Miettinen 10380.1 (H)	MN991176	MW045423
*Sistotremastrum aculeatum*	Cui 8401 (BJFC)	KX081133	KX081184
*Sistotremastrum aculeocrepitans*	KHL 16097 (URM)	MN937564	MN937564
*Sistotremastrum confusum*	KHL 16004 (URM)	MN937567	MN937567
*Sistotremastrum denticulatum*	Motato-Vásquez 894 (SP, holotype)	MN954694	MW045424
*Sistotremastrum fibrillosum*	LIP 0001413 (holotype)	NR_161047	NG_075239
*Sistotremastrum fibrillosum* s. l.	GUY13-119 (GG)	MG913224	MG913210
*Sistotremastrum fibrillosum* s. l.	KHL 16988 (MG)	MN937568	MN937568
*Sistotremastrum geminum*	Miettinen 14333 (MAN, holotype)	MN937568	MN937568
*Sistotremastrum induratum*	Spirin 8598 (H, holotype)	MT002324	MT664173
*Sistotremastrum mendax*	KHL 12022 (O, holotype)	MN937570	MN937570
*Sistotremastrum rigidum*	Motato-Vásquez 833 (SP, holotype)	MN954693	MW045435
*Sistotremastrum suecicum*	Kunttu 5959 (H)	MT075859	MT002335
*Sistotremastrum suecicum*	Miettinen 14550.1 (H)	MT075860	MT002336
*Sistotremastrum suecicum*	KHL 11849 (GB)	MN937571	MN937571
*Sistotremastrum vigilans*	Fonneland 2011-78 (O, holotype)	MN937572	MN937572
*Sistotremastrum vigilans*	Spirin 8778 (H)	MN991182	MN991182
*Subulicystidium tropicum*	He 3968 (BJFC)	MK204531	MK204544
*Suillosporium cystidiatum*	Spirin 3830 (H)	MN937573	MN937573
*Trechispora alnicola*	AFTOL-ID 665	DQ411529	AY635768
*Trechispora araneosa*	KHL8570 (GB)	AF347084	AF347084
*Trechispora bambusicola*	CLZhao 3302 (SWFC)	MW544021	MW520171
*Trechispora bispora*	CBS 142.63 (holotype)	MH858241	MH869842
*Trechispora cohaerens*	TU 110332	UDB008249	–
*Trechispora cohaerens*	TU 115568	UDB016421	–
*Trechispora confinis*	KHL11064 (GB)	AF347081	AF347081
*Trechispora copiosa*	AMO456	MN701019	MN687976
*Trechispora copiosa*	AMO422 (holotype)	MN701013	MN687971
*Trechispora cyatheae*	FR-0219442	UDB024014	UDB024014
*Trechispora cyatheae*	FR-0219443 (holotype)	UDB024015	UDB024015
***Trechispora dentata***	**Dai 22565 (BJFC)**	**OK298491** *	**OM049408** *
***Trechispora dimitiella***	**Dai 21931 (BJFC)**	**OK298492** *	**OK298948** *
***Trechispora dimitiella***	**Dai 21181 (BJFC)**	**OK298493** *	**OK298949** *
*Trechispora echinocristallina*	FR-0219445 (holotype)	UDB024018	UDB024019
*Trechispora echinocristallina*	FR-0219448	UDB024022	–
*Trechispora echinospora*	MA-Fungi 82485 (holotype)	JX392845	JX392846
*Trechispora farinacea*	KHL 8793 (GB)	AF347089	AF347089
*Trechispora farinacea*	KHL 8451 (GB)	AF347082	AF347082
*Trechispora fimbriata*	CLZhao 7969 (SWFC)	MW544024	MW520174
*Trechispora fimbriata*	CLZhao 4154 (SWFC, holotype)	MW544023	MW520173
*Trechispora fissurata*	CLZhao 4571 (SWFC, holotype)	MW544027	MW520177
*Trechispora fissurata*	CLZhao 995 (SWFC)	MW544026	MW520176
***Trechispora fragilis***	**Dai 20535 (BJFC)**	**OK298494** *	**OK298950** *
*Trechispora gelatinosa*	AMO1139 (holotype)	MN701021	MN687978
*Trechispora gelatinosa*	AMO824	MN701020	MN687977
*Trechispora havencampii*	SFSU DED8300 (holotype)	NR_154418	NG_059993
*Trechispora hymenocystis*	TL 11112 (holotype)	UDB000778	UDB000778
*Trechispora hymenocystis*	KHL 8795 (GB)	AF347090	AF347090
*Trechispora incisa*	GB0090648	KU747095	KU747087
*Trechispora incisa*	GB0090521	KU747093	–
*Trechispora kavinioides*	KGN 981002 (GB)	AF347086	AF347086
***Trechispora laevispora***	**Dai 21655 (BJFC)**	**OK298495** *	**OM108710**
*Trechispora minispora*	MEXU 28300 (holotype)	MK328886	MK328894
*Trechispora minispora*	MEXU 28301	MK328886	MK328895
*Trechispora mollis*	URM 85884 (holotype)	MK514945	MH280003
*Trechispora mollusca*	DLL2011-186 (CFMR)	KJ140681	–
*Trechispora mollusca*	DLL2010-077 (CFMR)	JQ673209	–
*Trechispora nivea*	GB0102694	KU747096	AY586720
*Trechispora nivea*	MA-Fungi 74044	JX392832	JX392833
*Trechispora papillosa*	AMO713	MN701022	MN687979
*Trechispora papillosa*	AMO795 (holotype)	MN701023	MN687981
*Trechispora regularis*	KHL10881 (GB)	AF347087	AF347087
*Trechispora rigida*	URM 85754	MT406381	MH279999
*Trechispora* sp.	AMO799	MN701008	MN687969
*Trechispora* sp.	AMO440	MN701006	MN687967
*Trechispora* sp.	KHL16968 (O)	MH290763	MH290763
***Trechispora* sp.**	**Dai 22173 (BJFC)**	**OK298496** *	**OK298951** *
***Trechispora* sp.**	**Dai 22174 (BJFC)**	**OK298497** *	**OK298952** *
*Trechispora stevensonii*	TU 115499	UDB016467	UDB016467
*Trechispora stevensonii*	MA-Fungi 70669	JX392841	JX392842
*Trechispora subsphaerospora*	KHL 8511 (GB)	AF347080	AF347080
*Trechispora termitophila*	AMO396 (holotype)	MN701025	MN687983
*Trechispora termitophila*	AMO390	MN701024	MN687982
*Trechispora torrendii*	URM 85886 (holotype)	MK515148	MH280004
*Tubulicium raphidisporum*	He 3191 (BJFC)	MK204537	MK204545

*Newly generated sequences for this study. New species and new combinations or putatively new species are in bold.

The MP analysis is applied to the ITS data set sequences. Approaches to phylogenetic analysis follow Liu and Dai (2021), and the tree construction procedure is performed in PAUP* version 4.0 beta 10 software (Swofford, 2002). All characters are equally weighted, and gaps are treated as missing data. Trees are inferred using the heuristic search option with tree bisection and reconnection (TBR) branch swapping, and 1,000 random sequence additions maxtrees are set to 5,000, branches of zero length are collapsed, and all parsimonious trees are saved. Clade robustness is assessed using a bootstrap (BT) analysis with 1,000 replicates (Felsenstein, 1985). Descriptive tree statistics tree length (TL), consistency index (CI), retention index (RI), rescaled consistency index (RC), and homoplasy index (HI) are calculated for each maximum parsimonious tree (MPT) generated.

Maximum likelihood research is conducted with RAxML-HPC v. 8.2.3 (Stamatakis, 2014) and RAxML-HPC through the CIPRES Science Gateway (Miller et al., 2009^2^). Statistical support values (BS) are obtained using nonparametric bootstrapping with 1,000 replicates. The BI analysis is performed with MrBayes 3.2.7a (Ronquist and Huelsenbeck, 2003). Four Markov chains are run for two runs from random starting trees for 4 million generations (ITS) and 8 million generations (5.8S + nLSU) until the split deviation frequency value reaches <0.01, and trees are sampled every 1,000 generations. The first 25% of the sampled trees are discarded as burn-in, and the remaining ones are used to reconstruct a majority rule consensus tree and to calculate Bayesian posterior probabilities (BPP) of the clades.

The optimal substitution models for the combined data set are determined using the Akaike information criterion (AIC) implemented in MrModeltest 2.3 (Posada and Crandall, 1998; Nylander, 2004) after scoring 24 models of evolution by PAUP* version 4.0 beta 10 software (Swofford, 2002). The selected model applied in the BI analyses and ML analyses is the model GTR + I + G.

Branches that received BT support for ML (BS), MP (BP), and BPP greater than 65% (BS), 70% (BP), and 0.9 (BPP) are considered as significantly supported, respectively. Additionally, the ML analysis results in the best tree, and only the ML tree is presented along with the support values from the MP and BI analyses. FigTree v1.4.4 (Rambaut, 2018) is used to visualize the resulting tree.

### Divergence Time Estimation

Divergence time is estimated with the BEAST v2.6.5 software package (Bouckaert et al., 2019) with 5.8S and nLSU sequences representing all main lineages in Basidiomycota (Table 2). Sequences of the species are adopted partly from the topology established by Wang et al. (2021). *Neurospora crassa* Shear and B.O. Dodge from Ascomycota are designated as outgroup taxon (Wang et al., 2021). A BEAST XML input file is generated with BEATUti v2. The estimation of rates of evolutionary changes at nuclear acids is using ModelTest 3.7 with the GTR substitution model (Posada and Crandall, 1998). A log-normal distribution is employed for molecular clock analysis (Drummond and Rambaut, 2007). A Yule speciation model is selected as prior assuming a constant speciation rate per lineage. Three fossil fungi, *viz. Paleopyrenomycites devonicus* (Taylor et al., 1999, 2005), *Archaeomarasmius leggetti* (Hibbett et al., 1995, 1997), and *Quatsinoporites cranhamii* (Smith et al., 2004; Berbee and Taylor, 2010) are taken from Wang et al.’s (2021) study. An XML file is conducted for 10 billion generations, producing log files and trees files. The log file is analyzed in Tracer 1,^3^ and a maximum clade credibility (MCC) tree is interpreted in TreeAnnotator by trees file, removing the first 10% of the sampled trees as burn-in, and viewed in FigTree v1.4.2.

**TABLE 2:** Information of taxa used in molecular clock analysis.

Species	Specimen no.	ITS	nLSU
*Amylocorticium cebennense*	HHB-2808	GU187505	GU187561
*Anomoloma myceliosum*	MJL-4413	GU187500	GU187559
*Athelia arachnoidea*	CBS 418.72	GU187504	GU187557
*Auricularia heimuer*	Xiaoheimao	LT716074	KY418890
*Auricularia* sp.	PBM 2295	DQ200918	AY634277
*Australovuilleminia coccinea*	MG75	HM046875	HM046931
*Boletopsis leucomelaena*	AFTOL-ID 1527	DQ484064	DQ154112
*Bondarzewia montana*	AFTOL-ID 452	DQ200923	DQ234539
*Brevicellicium atlanticum*	LISU 178566	NR_119820	HE963774
*Brevicellicium atlanticum*	LISU 178590	HE963775	HE963776
*Brevicellicium daweishanense*	CLZhao 17860	MW302337	MW293866
*Brevicellicium daweishanense*	CLZhao 18255	MW302338	MW293867
*Brevicellicium exile*	MA-Fungi 26554	HE963777	HE963778
*Brevicellicium olivascens*	KHL8571	HE963792	HE963793
*Brevicellicium olivascens*	MA-Fungi 23496	HE963787	HE963788
*Brevicellicium xanthum*	CLZhao 17781	MW302340	MW293869
*Brevicellicium xanthum*	CLZhao 2632	MW302339	MW293868
*Bridgeoporus sinensis*	Cui 10013	KY131832	KY131891
*Calocera cornea*	AFTOL-ID 438	AY789083	AY701526
*Coltricia perennis*	Cui 10319	KU360687	KU360653
*Coltriciella dependens*	Dai 10944	KY693737	KY693757
*Corticium roseum*	MG43	GU590877	AY463401
*Craterocolla cerasi*	TUB 020203	KF061265	KF061265
*Cryptococcus humicola*	AFTOL-ID 1552	DQ645516	DQ645514
*Dacryopinax spathularia*	AFTOL-ID 454	AY854070	AY701525
*Dextrinocystis calamicola*	He 5700	MK204534	MK204547
*Dextrinocystis calamicola*	He5693	MK204533	MK204546
*Exidia recisa*	EL 15-98	AF347112	AF347112
*Exidiopsis calcea*	MW 331	AF291280	AF291326
*Fasciodontia brasiliensis*	MSK-F 7245a	MK575201	MK598734
*Fasciodontia bugellensis*	MSK-F 5548	MK575204	MK598736
*Fibrodontia alba*	TNMF 24944	KC928274	KC928275
*Fibrodontia gossypina*	AFTOL-ID 599	DQ249274	AY646100
*Fomitiporia hartigii*	MUCL 53551	JX093789	JX093833
*Fomitiporia mediterranea*	AFTOL 688	AY854080	AY684157
*Gloeophyllum sepiarium*	Wilcox-3BB	HM536091	HM536061
*Gloeophyllum striatum*	ARIZAN 027866	HM536092	HM536063
*Grifola frondosa*	AFTOL-ID 701	AY854084	AY629318
*Gymnopilus picreus*	ZRL2015011	LT716066	KY418882
*Hymenochaete rubiginosa*	He1049	JQ716407	JQ279667
Hyphodontia densispora	LWZ 20170908-5	MT319426	MT319160
*Hyphodontia zhixiangii*	LWZ 20170818-13	MT319420	MT319151
*Jaapia argillacea*	CBS 252.74	GU187524	GU187581
*Gomphidius roseus*	MB 95-038	DQ534570	DQ534669
*Kneiffiella barba-jovis*	KHL 11730	DQ873609	DQ873610
*Kneiffiella subalutacea*	LWZ 20170816-9	MT319407	MT319139
*Lepiota cristata*	ZRL20151133	LT716026	KY418841
*Leptosporomyces raunkiaeri*	HHB-7628	GU187528	GU187588
*Leucophellinus hobsonii*	Cui 6468	KT203288	KT203309
*Lyomyces macrosporus*	LWZ20170817-2	MT319459	MT319194
*Multiclavula mucida*	AFTOL-ID 1130	DQ521417	AY885163
*Neoantrodiella gypsea*	Cui 10372	KT203290	MT319396
*Neoantrodiella thujae*	Dai 5065	KT203293	*MT319397*
*Neurospora crassa*	OR74A	HQ271348	AF286411
*Nigrofomes melanoporus*	JV 1704/39	MF629835	MF629831
*Nigrofomes sinomelanoporus*	Cui 5277	MF629836	MT319398
*Porodaedalea chinensis*	Cui 10252	KX673606	MH152358
*Porpomyces mucidus*	Dai 12692	KT157833	KT157838
*Porpomyces submucidus*	Cui 5183	KU509521	KT152145
*Pteridomyces galzinii*	GB0150230	LR694188	LR694210
*Pteridomyces galzinii*	Bernicchia8122	MN937559	MN937559
*Ramaria rubella*	AFTOL-ID 724	AY854078	AY645057
*Rigidoporus corticola*	ZRL20151459	LT716075	KY418899
*Rigidoporus ginkgonis*	Cui 5555	KT203295	KT203316
*Scytinopogon angulisporus*	TFB13611	–	JQ684661
*Scytinopogon pallescens*	He 5192	–	MK204553
*Sertulicium chilense*	MA-Fungi 86368	HG315521	–
*Sertulicium granuliferum*	He 3338	MK204552	MK204540
*Sertulicium jacksonii*	Spirin 10425	MN987943	MN987943
*Sertulicium lateclavigerum*	LY 13467	MG913225	–
*Sertulicium limonadense*	LIP 0001683	MT180981	MT180978
*Sertulicium niveocremeum*	KHL13727	MN937563	MN937563
*Sertulicium vernale*	Soderholm 3886	MT002311	MT664174
*Sistotremastrum aculeatum*	Cui 8401	KX081133	KX081184
*Sistotremastrum aculeatum*	Miettinen 10380.1	MN991176	MW045423
*Sistotremastrum aculeocrepitans*	KHL 16097	MN937564	MN937564
*Sistotremastrum confusum*	KHL 16004	MN937567	MN937567
*Sistotremastrum denticulatum*	MV894	MN954694	MW045424
*Sistotremastrum fibrillosum*	LIP 0001413	NR_161047	NG_075239
*Sistotremastrum fibrillosum* s. l.	GUY13-119	MG913224	MG913210
*Sistotremastrum fibrillosum* s. l.	KHL 16988	MN937568	MN937568
*Sistotremastrum geminum*	Miettinen 14333	MN937568	MN937568
*Sistotremastrum induratum*	Spirin 8598	MT002324	MT664173
*Sistotremastrum mendax*	KHL12022	MN937570	MN937570
*Sistotremastrum rigidum*	MV833	MN954693	MW045435
*Sistotremastrum suecicum*	Kunttu 5959	MT075859	MT002335
*Sistotremastrum suecicum*	Miettinen 14550.1	MT075860	MT002336
*Sistotremastrum suecicum*	KHL 11849 (GB)	MN937571	MN937571
*Sistotremastrum vigilans*	Fonneland 2011-78	MN937572	MN937572
*Sistotremastrum vigilans*	Spirin 8778	MN991182	MN991182
*Subulicystidium tropicum*	He3968	MK204531	MK204544
*Suillosporium cystidiatum*	VS3830	MN937573	MN937573
*Suillus pictus*	AFTOL 717	AY854069	AY684154
*Thelephora ganbajun*	ZRL20151295	LT716082	KY418908
*Trametes versicolor*	ZRL20151477	LT716079	KY418903
*Trechispora hymenocystis*	KHL8795	AF347090	AF347090
*Tremellodendron* sp.	PBM2324	DQ411526	–
*Tubulicium raphidisporum*	He 3191	MK204537	MK204545
*Ustilago maydis*	AFTOL 505	AY854090	AF453938
*Xylodon heterocystidiatus*	LWZ 20171015-33	MT319518	MT319264

## Results

### Phylogenetic Analyses

**FIGURE 1:**
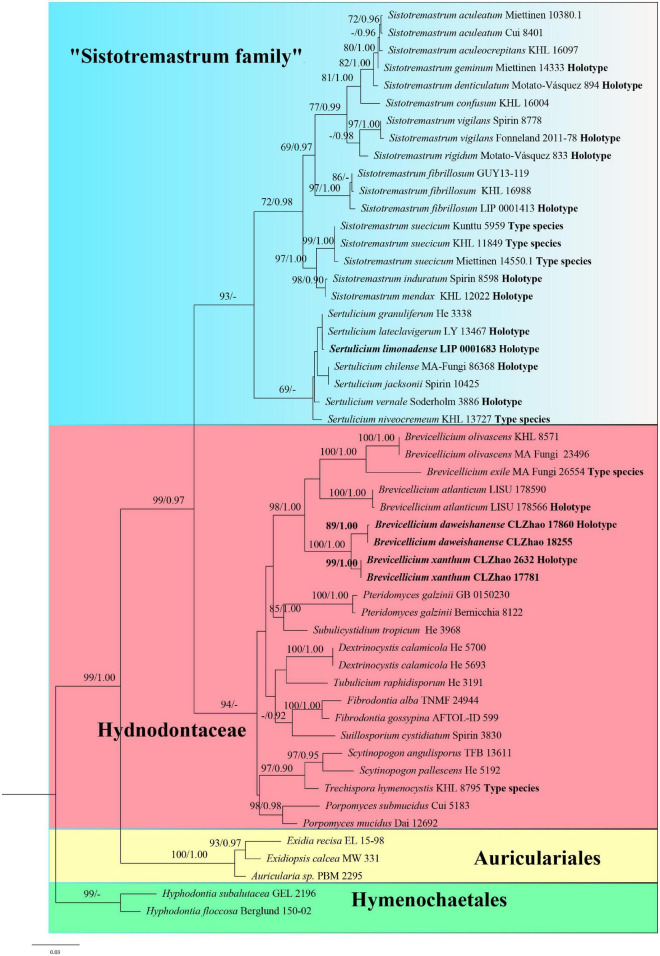
Phylogeny of Trechisporales generated by maximum likelihood (ML) analyses based on combined 5.8S + nLSU sequences. Branches are labelled with ML bootstrap (BT) >65%, and Bayesian posterior probabilities (BPP) >0.90, respectively. New combinations, the sequence origin from holotype and the type status of the species in the genus are indicated in bold.

The concatenated 5.8S + nLSU data set contains 50 5.8S and 50 nLSU sequences from 52 fungal specimens representing 35 taxa in Trechisporales. The data set has an aligned length of 1,528 characters, of which 1,126 are constant, 89 are variable but parsimony-uninformative, and 313 are parsimony-informative. The average standard deviation (SD) of split frequencies is 0.005271 (BI). Three new combinations, namely, *Brevicellicium daweishanense*, *Brevicellicium xanthum*, and *Sertulicium limonadense*, are proposed based on the examination of type materials and phylogenetic analyses of type sequences (Figure 1).

**FIGURE 2:**
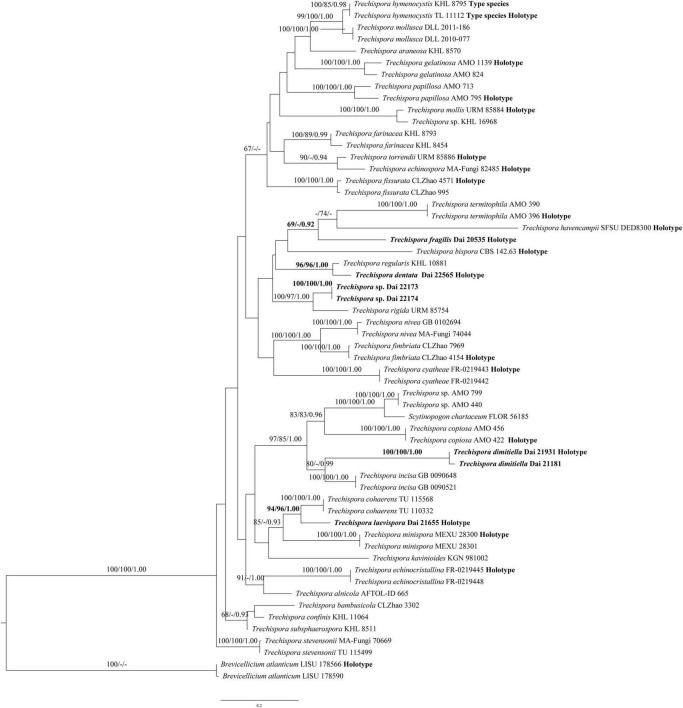
Phylogeny of *Trechispora* generated by ML analyses based on combined ITS sequences. Branches are labelled with ML BT >65%, Parsimony Bootstrap Proportions >70%, and BPP >0.90, respectively. Putatively new species, the sequence origin from the holotype, and the type status of the species in the genus are indicated in bold.

The ITS data set contains sequences from 58 fungal specimens representing 36 *Trechispora* taxa (4 new species and another 32 *Trechispora* taxa). The data set has an aligned length of 753 characters, of which 284 are constant, 72 are variable but parsimony-uninformative, and 397 are parsimony-informative. MP analysis yields 13 equally parsimonious trees (TL = 2,318, CI = 0.398, RI = 0.638, RC = 0.254, and HI = 0.602). The average SD of split frequencies in BI analyses is 0.006959 (BI). The phylogenetic tree (Figure 2) reveals four new and independent lineages represented by our specimens, indicating that they are phylogenetically distinct from the species currently known in the genus. In addition, another taxon (Dai 22173 and Dai 22174) is treated as *Trechispora* sp.

**FIGURE 3:**
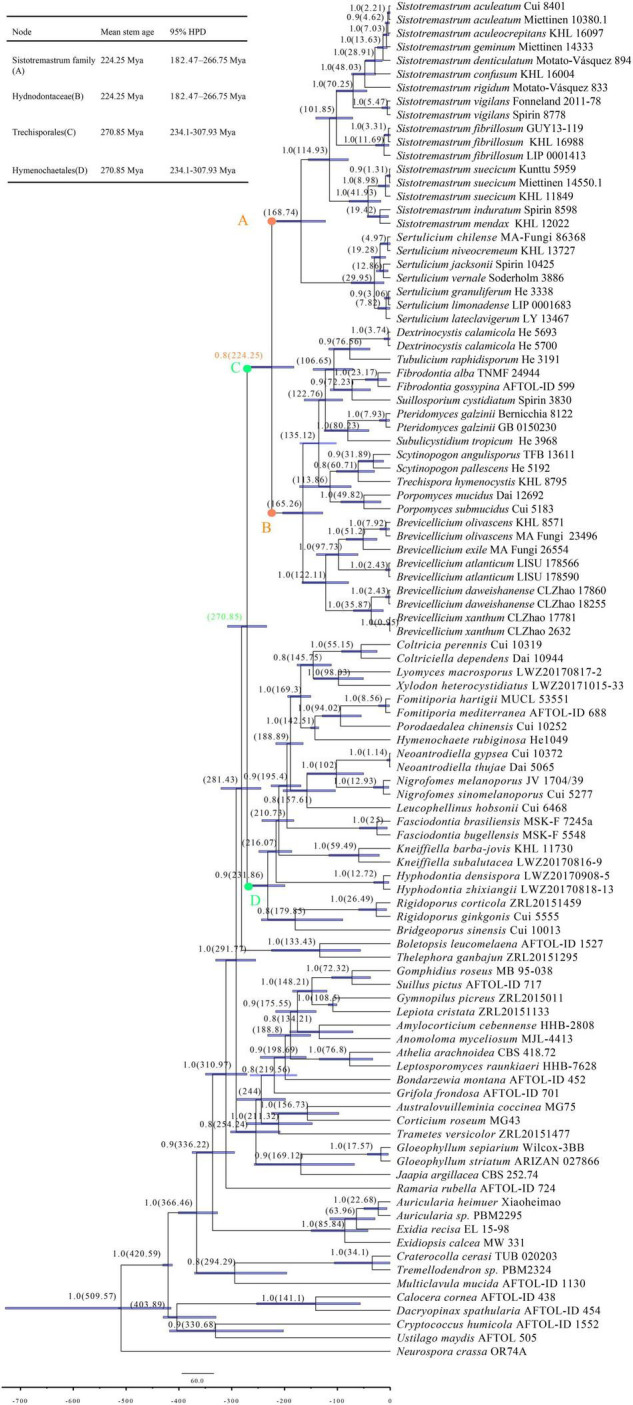
Maximum clade credibility (MCC) chronogram and estimated divergence times of all main lineages in Basidiomycota inferred from the combined data set of 5.8S and LSU regions. The estimated divergence times of 95% highest posterior density (HPD) for all clades are indicated as node bars. The colored dots refer to the positions of the mean stem age of *Sistotremastrum* family, Hydnodontaceae, Trechisporales, and Hymenochaetales. The BPP above 0.8 and the mean divergence times of clades are labelled above and below the branches, respectively, at the nodes.

The combined data set for the molecular clock analysis includes 100 collections, of which 47 belonged to Trechisporales. This data set results in a concatenated alignment of 1,588 characters with GTR as the best-fit evolutionary model. The MCC tree is used to study divergence time. The tree shows that Trechisporales occurs in a mean stem age of 270.85 Mya with a 95% highest posterior density (HPD) of 234.1–307.93 Mya (Figure 3). The tree also shows that the *Sistotremastrum* family and Hydnodontaceae occur in a mean stem age of 224.25 Mya [posterior probabilities (PP) = 0.8] with a 95% HPD of 182.47–266.75 Mya.

### Taxonomy

***Sistotremastrum* family**

“Type genus”: *Sistotremastrum* J. Erikss.

*Habitat*: It grows on rotten angiosperm and gymnosperm wood.

Basidioma are resupinate, thin, pruinose, or waxy. Hymenophores are smooth, verruculose, or odontioid-semiporoid. The hyphal structure is monomitic; generative hyphae bear clamp connections, CB(+). Cystidia and hyphidia are present in some species. Basidia are clavate or cylindrical, often with a median constriction, mostly with 2–4 or 4–6 sterigmata, and rarely with 6–8 sterigmata. Basidiospores are narrowly ellipsoid, ovoid, or cylindrical, thin-walled (but the wall is distinct), smooth, inamyloid, and acyanophilous.

*Notes*: *Sistotremastrum* family accommodates the genera *Sistotremastrum* and *Sertulicium* in the order Trechisporales based on its distinct lineage in the phylogenetic analysis. The combined phylogeny of two-gene data (Figure 1) demonstrates that *Sistotremastrum* family forms a supported sister clade to Hydnodontaceae. Basidia of most species in the *Sistotremastrum* family have more than four sterigmata, and basidiospores are smooth, while basidia of species in Hydnodontaceae have four sterigmata and their basidiospores are smooth to verrucose or aculeate. In addition, ampullate septa are only present in *Scytinopogon*, *Trechispora*, and *P. mucidus* in Hydnodontaceae.

**FIGURE 4:**
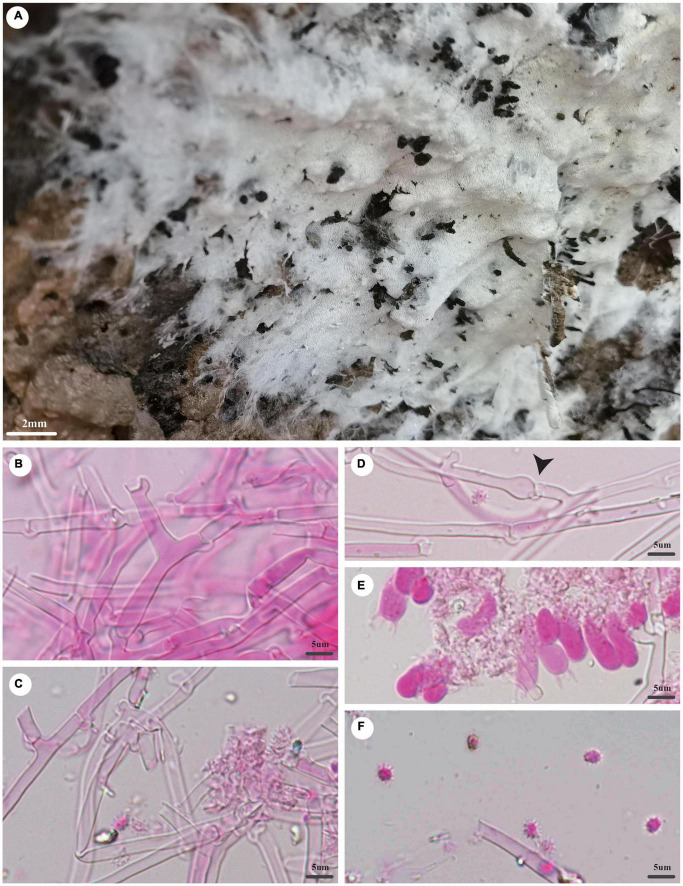
*Trechispora dentata* (holotype, Dai 22565). **(A)** A basidioma, **(B)** hyphae from subiculum, **(C)** hyphae from trama, **(D)** hyphae with ampullate septa (black arrow), **(E)** basidia and basidioles, and **(F)** basidiospores. Photo by Ya-Ping Lian and Zhan-Bo Liu.

***Trechispora dentata*** Z.B. Liu and Yuan Yuan, sp. November Figure 4

*MycoBank number*: MB 842865.

*Type*: China, Yunnan province, Sipsongpanna, Mengla County, XiShuangBanNa Tropical Botanical Garden, on soil, in southwestern China, ca. E 101° 25′, N 21° 41′, alt. 570 m. The vegetation is a natural tropical forest. 4 July 2021, Y.C. Dai 22565 (holotype BJFC 037139).

*Etymology*: *Dentata* (Lat.): It refers to the species having a dentate hymenophore.

*Basidioma*: They are annual, resupinate, soft when fresh, fragile when dry, easily separable from the substratum, up to 2.5-cm long, 2-cm wide, and less than 1-mm thick at the center; hymenial surface irpicoid, white when fresh, becoming cream (4A2/3) when dry; margin indistinct and fimbriate, mycelial cords absent; pores or aculei 3–4/mm; hymenophore lacerate to dentate; subiculum very thin to almost absent; tubes or aculei concolorous with a hymenial surface, less than 1 mm long.

*Hyphal structure*: Hyphal system is monomitic; generative hyphae bear clamp connections; ampullate septa occasionally present in subiculum and trama, up to 5-μm wide; all hyphae IKI−, CB− are unchanged in KOH; rhomboidal calcium oxalate crystals are scattered.

*Subiculum*: Generative hyphae hyaline, thin- to thick-walled, frequently branched, loosely interwoven, 2–4 μm in diameter.

*Tubes or aculei*: Generative hyphae in trama hyaline, thin- to thick-walled, frequently branched, loosely interwoven, 2–3 μm in diameter; cystidia and cystidioles are absent; basidia are clavate or barrel-shaped, hyaline, bearing four sterigmata and a basal clamp connection, 10–15 × 4–5 μm; basidioles are similar to basidia in shape but slightly shorter.

*Basidiospores*: They are ellipsoid, hyaline, thick-walled, aculeate, occasionally with one guttule, IKI−, CB−, (4−)4.1–5 × (3−)3.2–4(−4.1) μm (including ornamentation), *L* = 4.46 μm, *W* = 3.66 μm, *Q* = 1.22 (*n* = 60/1); (2.2−)2.6–3.7(−3.8) × 2–2.5 μm (excluding ornamentation), *L*′ = 3.17 μm, *W*′ = 2.23 μm, and *Q*′ = 1.42 (*n* = 60/1).

*Notes*: *T. dentata* was discovered in the Yunnan Province of China. Phylogenetically, *T. dentata* is close to *Trechispora regularis* (Murrill) Liberta with strong support (96% BS, 96% BP, 1.00 BPP; Figure 2). However, *T. regularis* is strictly poroid (Liberta, 1973), and basidiospores of *T. dentata* are smaller than that of *T. regularis* [4.1–5 × 3.2–4 μm vs. 4–5.5 × 3.5–5 μm in *T. regularis* (including ornamentation); Liberta, 1973].

**FIGURE 5:**
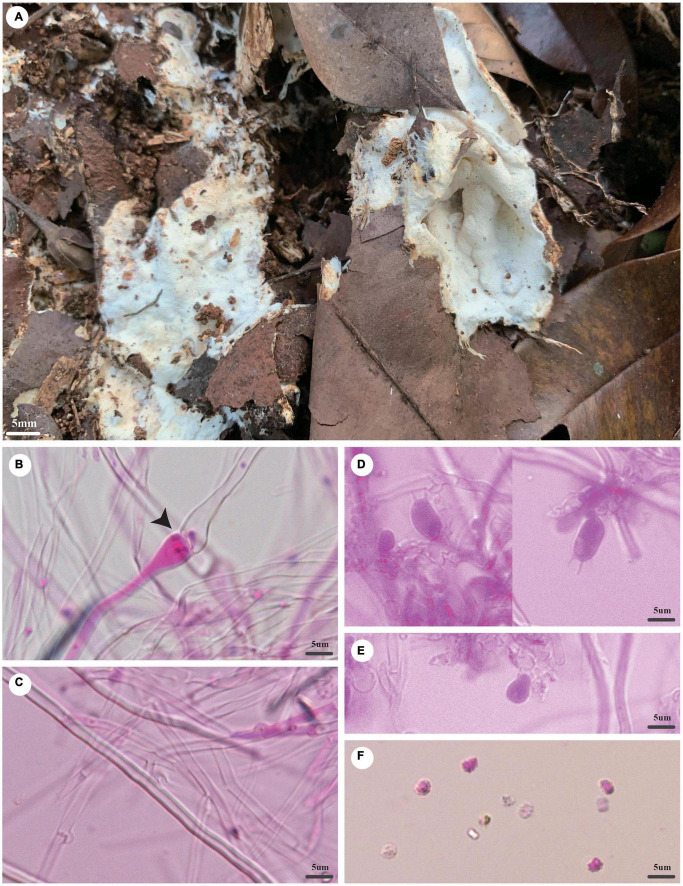
*Trechispora dimitiella* (holotype, Dai 21931). **(A)** A basidioma, **(B)** hyphae with ampullate septa from subiculum (black arrow), **(C)** hyphae from tubes, **(D)** basidia, **(E)** basidioles, and **(F)** basidiospores. Photo by Ya-Ping Lian and Zhan-Bo Liu.

***Trechispora dimitiella*** Z.B. Liu and Yuan, sp. November Figure 5

*MycoBank number*: MB 842866.

*Type*: China, Hainan Province, Haikou, Jinniuling Park, on a rotten leaf, in southwestern China, ca. E 110° 19′, N 20° 1′, alt. 17 m. The vegetation is a plantation in tropical China. 7 November 2020, Y.C. Dai 21931 (holotype BJFC 035830).

*Etymology*: *Dimitiella* (Lat.): It refers to the species having a dimitic hyphal system.

*Basidioma*: They are annual, resupinate, soft when fresh, fragile when dry, easily separable from the substratum, up to 6-cm long, 4-cm wide, and approximately 3-mm thick at the center; the hymenial surface is poroid, pore surface white to cream (4A2/3) when fresh, becoming white to buff-yellow (4A4) when dry; margin indistinct, often with emerging mycelial cords; pores angular, 5–6/mm; dissepiments thin, lacerate; subiculum up to 1 mm thick; tubes concolorous with a poroid surface, up to 2 mm long.

*Hyphal structure*: Hyphal system is dimitic; generative hyphae bear clamp connections; ampullate septa occasionally present in subiculum and trama, up to 4.5 μm wide; all hyphae IKI−, CB− are unchanged in KOH; rhomboidal calcium oxalate crystals are scattered.

*Subiculum*: Generative hyphae hyaline, thin-walled, rarely branched, 2–3 μm in diameter; skeletal hyphae thick-walled with a wide lumen, unbranched, loosely interwoven, 2–4 μm diameter.

*Tubes*: Generative hyphae hyaline, thin-walled, rarely branched, 1.5–2.5 μm in diameter; skeletal hyphae thick-walled with a wide lumen, unbranched, loosely interwoven, 2–3 μm in diameter; cystidia and cystidioles are absent; basidia are barrel-shaped, hyaline, bearing four sterigmata and a basal clamp connection, 9.5–12 × 4–5 μm; basidioles are similar to basidia in shape but slightly shorter.

*Basidiospores*: They are ellipsoid, hyaline, thick-walled, aculeate, IKI−, CB−, (3.5−)3.6–4(−4.2) × (2.5–)2.7–3.1(−3.2) μm (including ornamentation), *L* = 3.84 μm, *W* = 2.92 μm, *Q* = 1.31–1.33 (*n* = 60/2); (2.6−)2.7–3.4(−3.7) × 2–2.6(−2.9) μm (excluding ornamentation), *L*′ = 3.04 μm, *W*′ = 2.18 μm, and *Q*′ = 1.38–1.4 (*n* = 60/2).

Additional specimen examined (paratypes): China, Yunnan Province, Jinghong, Primeval Forest Park, on soil, 7 July 2021, Y.C. Dai 22601 (BJFC), Dai 22602 (BJFC). Malaysia, Selangor, Kota Damansara, Community Forest Reserve, on rotten angiosperm wood, 7 December 2019, Y.C. Dai 21181 (BJFC 032835).

*Notes*: *T. dimitiella* was discovered in China and Malaysia. Most species in *Trechispora* are corticioid fungi with a monomitic hyphal structure, but *T. dimitiella* is different. Morphologically, *T. dimitiella* and *Trechispora brasiliensis* (Corner) K.H. Larss. share the poroid hymenophore with a dimitic hyphal system and aculeate basidiospores. However, the basidiospores of *T. dimitiella* are smaller than that of *T. brasiliensis* [3.6–4 × 2.7–3.1 μm vs. 4–4.5 × 3–4 μm in *T. brasiliensis* (including ornamentation), Larsson, 1992]. Phylogenetically, *T. dimitiella* is close to *Trechispora incisa* K.H Larss. (80% BS, 0.99 BPP; Figure 2), but *T. dimitiella* can be easily distinguished from *T. incisa* due to its poroid hymenophore with a dimitic hyphal system because *T. incisa* has arachnoid to farinose or minutely granulose hymenophore with a monomitic hyphal system (Larsson, 1996).

**FIGURE 6:**
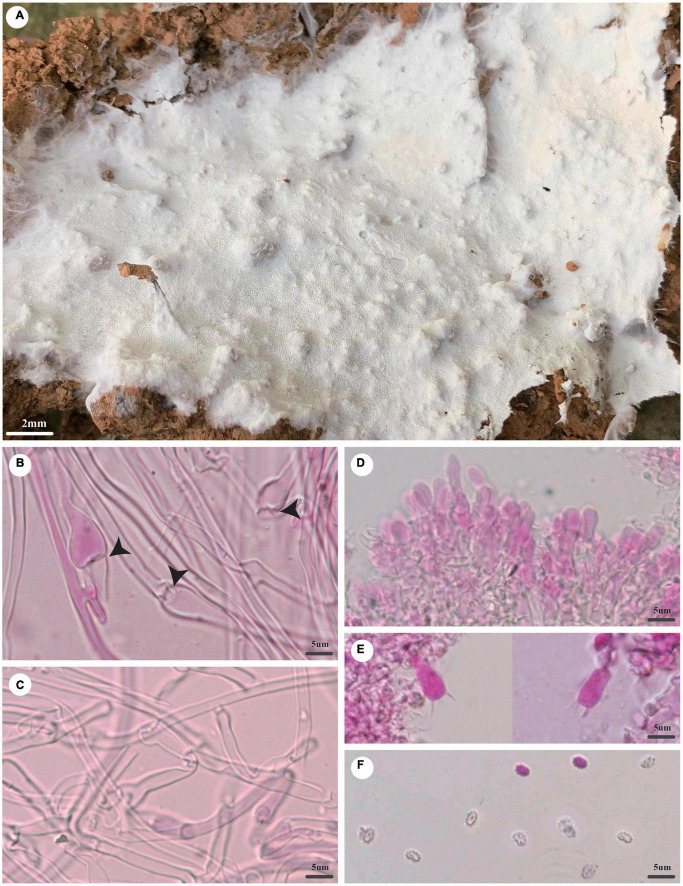
*Trechispora fragilis* (holotype, Dai 20535). **(A)** A basidioma, **(B)** hyphae with ampullate septa from subiculum (black arrows), **(C)** hyphae from aculei, **(D)** hymenium with basidioles, **(E)** basidia, and **(F)** basidiospores. Photo by Ya-Ping Lian and Zhan-Bo Liu.

***Trechispora fragilis*** Z.B. Liu and Yuan Yuan, sp. November Figure 6

*MycoBank number*: MB 842867.

*Type*: China, Yunnan Province, Sipsongpanna, Mengla County, XiShuangBanNa Tropical Botanical Garden, on the ground of the forest, in southwestern China, ca. E 101° 25′, N 21° 41′, alt. 570 m. The vegetation is a natural tropical forest. 18 August 2019, Y.C. Dai 20535 (holotype BJFC 032203).

*Etymology*: *Fragilis* (Lat.): It refers to the species having fragile basidiocarps.

*Basidioma*: They are annual, resupinate, soft when fresh, fragile when dry, easily separable from the substratum, up to 3 cm long, 2 cm wide, and less than 1 mm thick at the center; the hymenial surface is odontoid, white when fresh, becoming cream (4A2/3) to buff-yellow (4A4) when dry; margin is indistinct and fimbriate, often with emerging mycelial cords; aculei sparse, 4–6/mm; subiculum very thin to almost absent; aculei concolorous with a hymenial surface, less than 1 mm long.

*Hyphal structure*: Hyphal system monomitic; generative hyphae bear clamp connections; ampullate septa occasionally present in subiculum and aculei, up to 7 μm wide; all hyphae IKI−, CB− are unchanged in KOH; rhomboidal calcium oxalate crystals are scattered.

*Subiculum*: Generative hyphae hyaline, thin- to thick-walled, frequently branched, loosely interwoven, 1.5–4 μm in diameter.

*Aculei*: Generative hyphae in trama hyaline, thin- to thick-walled, frequently branched, loosely interwoven, 1.5–3 μm in diameter; cystidia and cystidioles are absent; basidia are clavate shaped, hyaline, bearing four sterigmata, and a basal clamp connection, 12–14 × 3.5–4 μm; basidioles are similar to basidia in shape but slightly shorter.

*Basidiospores*: Ellipsoid, hyaline, thick-walled, aculeate, IKI−, CB−, (3.2−)3.8–4(−4.2) × (2.4−)2.5–3 μm (including ornamentation), *L* = 3.53 μm, *W* = 2.79 μm, *Q* = 1.27 (*n* = 60/1); (2.6−)2.8–3.7(−4) × (1.9−)2–2.7(−3.1) μm (excluding ornamentation), *L*′ = 3.16 μm, *W*′ = 2.26 μm, and *Q*′ = 1.40 (*n* = 60/1).

*Notes*: *T. fragilis* was discovered in the Yunnan Province of China. Phylogenetically, *T. fragilis* groups with *Trechispora termitophila* Meiras-Ottoni and Gibertoni and *Trechispora havencampii* (Desjardin and B.A. Perry) Meiras-Ottoni and Gibertoni (69% BS, 0.92 BPP; Figure 2). *T. termitophila* can be easily distinguished from *T. fragilis* due to its coralloid basidioma. In addition, the basidiospores of *T. fragilis* are smaller than that of *T. termitophila* [6.5–7.5 μm vs. 4.5–5 μm in *T. termitophila* (including ornamentation), Meiras-Ottoni et al., 2021]. *T. havencampii* can also be easily distinguished from *T. fragilis* due to its coralloid basidioma. In addition, basidiospores of *T. fragilis* are smaller than that of *T. havencampii* [3.8–4 × 2.5–3 μm vs. 5.2–6.5 × 3.5–4.2 μm in *T. havencampii* (including ornamentation), Desjardin and Perry, 2015].

**FIGURE 7:**
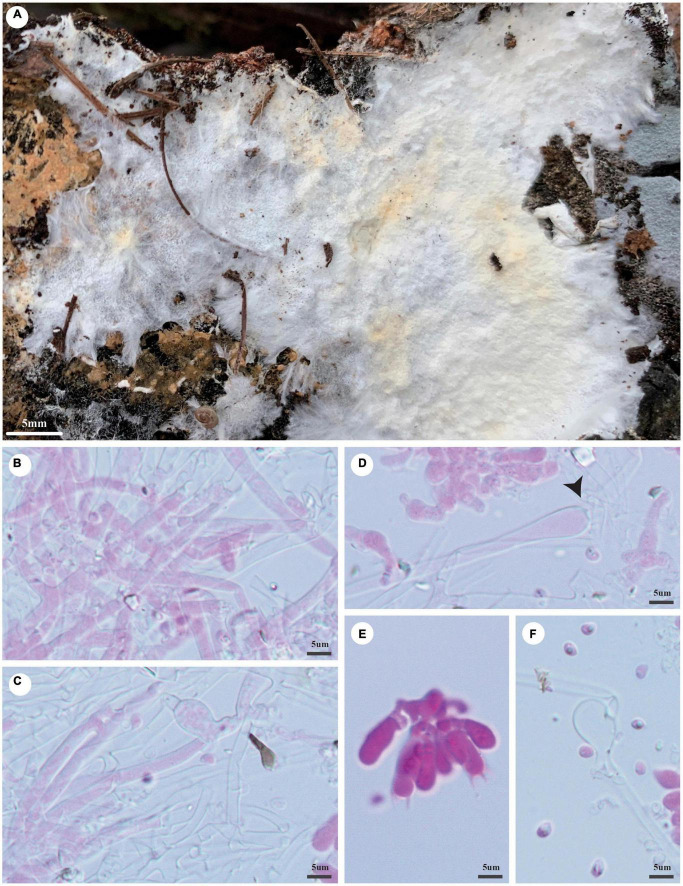
*Trechispora laevispora* (holotype, Dai 21655). **(A)** A basidioma, **(B,C)** hyphae from subiculum, **(D)** subicular hyphae with ampullate septa (black arrow) and a piece of hymenium, **(E)** basidia and basidioles, and **(F)** basidiospores. Photo by Ya-Ping Lian and Zhan-Bo Liu.

***Trechispora laevispora*** Z.B. Liu, Y.D. Wu and Yuan Yuan, sp. November Figure 7

*MycoBank number*: MB 842868.

*Type*: China, Inner Mongolia Autonomous Region, Arxan, Bailang Feng Scenic Spot, on the charred trunk of *Larix*, in southwestern China, ca. E 119° 56′, N 47° 10′, alt. 1,511 m. The vegetation is a natural boreal forest. 25 August 2020, Y.C. Dai 21655 (holotype BJFC 035556).

*Etymology*: *Laevispora* (Lat.): It refers to the species having smooth basidiospores.

*Basidioma*: They are annual, resupinate, soft when fresh and dry, up to 8 cm long, 3 cm wide, and less than 1 mm thick at the center; the hymenial surface is smooth, white when fresh and dry; margin is indistinct and fimbriate, often with emerging mycelial cords; subiculum very thin to almost absent.

*Hyphal structure*: Hyphal system monomitic; generative hyphae bear clamp connections; ampullate septa frequently present in subiculum and hymenium, up to 7 μm wide; all hyphae IKI−, CB− are unchanged in KOH; rhomboidal calcium oxalate crystals are abundant.

*Subiculum*: Generative hyphae hyaline, thin-walled, frequently branched, loosely interwoven, 1.5–3 μm in diameter.

*Hymenium*: Generative hyphae in subhymenium hyaline, thin-walled, frequently branched, 1.5–3 μm in diameter; cystidia and cystidioles are absent; basidia are clavate shaped, hyaline, bearing four sterigmata and a basal clamp connection, 11.5–15 × 4–5 μm; basidioles are similar to basidia in shape but slightly shorter.

*Basidiospores*: Ellipsoid, hyaline, thin-walled, smooth, IKI−, CB−, (2.5−) 2.6–3.2(−3.3) × (1.8−)1.9–2.2(−2.5) μm, *L* = 2.92 μm, *W* = 2.04 μm, and *Q* = 1.43 (*n* = 60/1).

*Notes*: *T. laevispora* was discovered in the Inner Mongolia Autonomous Region of China. Phylogenetically, *T. laevispora* groups with *Trechispora cohaerens* (Schwein.) Jülich and Stalpers with strong support (94% BS, 96% BP, 1.00 BPP; Figure 2). Both species share a smooth hymenophore, a monomitic hyphal system with smooth basidiospores. However, basidiospores of *T. cohaerens* are thick-walled and larger than that of *T. laevispora* (3.5–4 × 2.2–2.5 μm in *T. cohaerens*; Larsson, 1992).

***B. daweishanense*** (C.L. Zhao) Z.B. Liu and Yuan Yuan, comb. November

*MycoBank number*: MB 842869.

*Basionym*: *T. daweishanensis* C.L. Zhao, Phytotaxa 479(2): 153 (2021).

*Type*: China. Yunnan Province, Honghe, Pingbian County, Daweishan National Nature Reserve, on the fallen branch of angiosperms, 1 August 2019, CLZhao 17860 (holotype SWFC).

*Description*: See Zong et al. (2021, as *T. daweishanensis*).

***B. xanthum*** (C.L. Zhao) Z.B. Liu and Yuan Yuan, comb. November

*MycoBank number*: MB 842870.

*Basionym*: *T. xantha* C.L. Zhao, Phytotaxa 479(2): 155 (2021).

*Type*: China. Yunnan Province, Yuxi, Xinping County, Mopanshan National Forestry Park, on the trunk of *Albizia julibrissin*, 20 August 2017, CLZhao 2632 (holotype SWFC).

*Description*: See Zong et al. (2021, as *T. xantha*).

*Notes*: Zong et al. (2021) described *T. daweishanensis* and *T. xantha* as new species. However, in our phylogeny, they belong to the genus *Brevicellicium* (98% BS, 1.00 BPP; Figure 1). The type specimens of abovementioned species are studied [CLZhao 17860 (SWFC); CLZhao 2632 (SWFC)]. We do not observe ampullate hyphae from type materials as mentioned by Zong et al. (2021). We suppose that Zong et al. (2021) confused basidioles with ampullate hyphae (ampullate septa on some generative hyphae), which are remarkable characters of *Trechispora*. In fact, *T. daweishanensis* and *T. xantha* have a smooth hymenophore, a monomitic hyphal structure with clamped generative hyphae, and the absence of ampullate septa. They fit *Brevicellicium* well. Herein, we combine these two species in *Brevicellicium* based on morphological and phylogenetic evidence (Figure 1).

***S. limonadense*** (G. Gruhn and P. Alvarado) Z.B. Liu and Yuan Yuan, comb. November

*MycoBank number*: MB 842871.

*Basionym*: *S. limonadense* G. Gruhn and P. Alvarado, Phytotaxa 498(1): 36 (2021).

*Type*: French Guiana. On the bark of an unidentified dead trunk lying on the ground, October 22, 2013, LIP 0001683 (holotype).

*Description*: See Gruhn and Alvarado (2021, as *S. limonadense*).

*Notes*: Gruhn and Alvarado (2021) described *S. limonadense* as a new species. However, at the same time, Spirin et al. (2021) segregated the species around *S. niveocremeum* (Höhn. and Litsch.) J. Erikss. into the new genus *Sertulicium*. In our phylogeny, *S. limonadense* groups with *Sertulicium granuliferum* (Hallenb.) Spirin and Volobuev *Sertulicium lateclavigerum* (Boidin and Gilles) Spirin and Viner (Figure 1). We did not study specimens, but *S. limonadense* is characterized by smooth to tuberculate hymenophore and basidia have 6–8 sterigmata (Gruhn and Alvarado, 2021) and fits *Sertulicium* better. Hence, we transfer *S. limonadense* to *Sertulicium*.

## Discussion

Larsson (2007) showed that *S. suecicum* and *S. niveocremeum* (= *S. niveocremeum*) formed a strongly supported sister clade (94% BS, 1.00 BPP) to Hydnodontaceae within Trechisporales. However, in his phylogenetic analysis of 5.8S + nLSU, there were a few species in Hydnodontaceae and *Sistotremastrum* to establish a new family for *S. suecicum* and *S. niveocremeum.* Hence, Larsson (2007) named this clade *Sistotremastrum* family. The same strongly supported topology was recovered by Telleria et al. (2013); Gruhn et al. (2018), and Meiras-Ottoni et al. (2021) by the nLSU phylogenetic analysis. Spirin et al. (2021) presented a comprehensive study of *Sistotremastrum* and *Sertulicium* with 17 species. They used the nLSU region to perform phylogenetic analyses of 16 species in the two genera (Figure 1 in Spirin et al., 2021), except for *Sertulicium chilense* (Telleria, M. Dueñas and M.P. Martín) Spirin and Volobuev because the nLSU sequences of *S. chilense* were absent. However, they were not able to generate high support values for the node connecting *Sistotremastrum* and *Sertulicium* (87% BS, 0.87 BPP, Figure 1 in Spirin et al., 2021). As a result, they gave up establishing a new family too.

ITS1-5.8S-ITS2 is an important marker used for the barcoding of fungal species (Liu et al., 2021; Wangsawat et al., 2021). However, the difficulty in aligning ITS sequences for fungi in Trechisporales is evident because it is a data set covering taxa in distinct taxonomic levels (Larsson, 2007). Therefore, it is not a good idea to run combined analyses of ITS + nLSU, so we use the most stable and conservative portion of ITS (5.8S) and nLSU to our phylogenetic analyses of *Sistotremastrum* and *Sertulicium* (5.8S + nLSU) (Figure 1). We add *S. chilense* and *S. limonadense* to phylogenetic analyses. Our results of the *Sistotremastrum* are the same as phylogenetic analyses by Spirin et al. (2021, Figure 1). However, our phylogenetic analyses of *Sertulicium* are a bit different from that by Spirin et al. (2021, Figure 1) because the data sets used in both studies are different. Above all, we generate high support values for the node connecting *Sistotremastrum* and *Sertulicium* from ML analysis (93% BS) based on 5.8S and nLSU sequences; however, BI fails to provide support for the node (0.76 BPP).

Divergence time is estimated with 5.8S and nLSU sequences representing all main lineages in Basidiomycota (Figure 3). The MCC tree shows that Basidiomycota occurs in a mean stem age of 509.57 Mya. Trechisporales occurs in a mean stem age of 270.85 Mya. The tree also shows that the *Sistotremastrum* family and Hydnodontaceae occur in a mean stem age of 224.25 Mya (PP = 0.8). Zhao et al. (2017) indicate that the divergence times of Basidiomycota are 530 Mya (the mean stem age). He et al. (2019) indicate that the divergence times of Trechisporales and Hydnodontaceae are 259 Mya (the mean stem age). Our experimental results agree with them. In this paper, we update the divergence times of Trechisporales and Hydnodontaceae and define the divergence time of the *Sistotremastrum* family.

Bayesian phylogenetic inference fails to provide support for the node of *Sistotremastrum* and *Sertulicium*, so we use the term “*Sistotremastrum* family” for the two genera without a formal description of the new family. In the future, we will sequence additional DNA regions or whole genomes, for a more robust phylogenetic analysis.

At present, there are only two species in the *Sistotremastrum* family ever been recorded from China, i.e., *Sistotremastrum aculeatum* Miettinen and Viner (Cui 8401) and *S. granuliferum* (He 3338; CLZhao 5531, 9771). Recently, we collected a specimen from the Yunnan Province of China (He 6276), and its morphological and DNA data demonstrated the specimen is S. limonadense. The species is a new record in China, and we have uploaded ITS and nLSU sequences of the specimen (He 6276) to GenBank. Above all, we study all the Chinese specimens of species in the Sistotremastrum family seriously, and their morphology fits the descriptions of Gruhn and Alvarado (2021) and Spirin et al. (2021). We also collected a specimen from the Hainan Province of China (Dai 17696). The ITS (OK298490) region is different from *Sistotremastrum fibrillosum* G. Gruhn and P. Alvarado by 6%, and morphologically it is similar to *S. fibrillosum.* However, we only have a single specimen, so for the time being we regard Dai 17696 as *Sistotremastrum* sp.

In this article, we use the whole ITS region in analyses of *Trechispora* to visualize the genetic distances among new taxa and those already described. *T. dentata*, *T. dimitiella*, *T. fragilis*, and *T. laevispora* are described as new to science based on morphological characteristics and molecular evidence (Figure 2). Most of these new species are found in subtropical or tropical Asia and conform to the phenomenon that subtropical or tropical Asia harbors high taxonomic diversity for all wood-decaying fungi (Dai, 2012; Cui et al., 2019). We also collected two resupinate specimens (Dai 22173 and Dai 22174) from the Hainan Province of China. The morphology of the two specimens corresponds to the concept of *Trechispora* and forms a distinct lineage within the *Trechispora* clade (100% BS, 1.00 BPP; Figure 2). However, these specimens are sterile, so we regard Dai 22173 and Dai 22174 as *Trechispora* spp. temporarily here.

Molecular phylogenetic analyses in the present study show that *Brevicellicium* forms a monophyletic clade in which all *Brevicellicium* species are included (98% BS, 1.00 BPP; Figure 1). However, when we add sequences of *T. xantha* and *T. daweishanensis*, we find sequences of a two-species cluster with *Brevicellicium* with high support (100% BS, 1.00 BPP; Figure 1). We request and examine type specimens from Zhao and find *T. xantha* and *T. daweishanensis* corresponding to the concept of *Brevicellicium* and they should be transferred to the genus *Brevicellicium* (see the notes of *B. daweishanense*).

## Data Availability Statement

The datasets presented in this study can be found in online repositories. The names of the repository/repositories and accession number(s) can be found in the article/supplementary material.

## Author Contributions

Z-BL: design of the research, performance of the research, and writing and revising this manuscript. Z-BL, HZ, Y-PL, Y-RW, C-GW, and W-LM: data analysis and interpretation. Z-BL, YY, and Y-DW: a collection of the materials. All authors contributed to the article and approved the submitted version.

## Conflict of Interest

The authors declare that the research was conducted in the absence of any commercial or financial relationships that could be construed as a potential conflict of interest.

## Publisher’s Note

All claims expressed in this article are solely those of the authors and do not necessarily represent those of their affiliated organizations, or those of the publisher, the editors and the reviewers. Any product that may be evaluated in this article, or claim that may be made by its manufacturer, is not guaranteed or endorsed by the publisher.

## Footnotes

1
http://mafft.cbrc.jp/alignment/server/

2
http://www.phylo.org

3
http://beast.community/tracer
